# Enhanced Wnt/β-catenin and Notch signalling in the activated canine hepatic progenitor cell niche

**DOI:** 10.1186/s12917-014-0309-1

**Published:** 2014-12-31

**Authors:** Baukje A Schotanus, Hedwig S Kruitwagen, Ted SGAM van den Ingh, Monique E van Wolferen, Jan Rothuizen, Louis C Penning, Bart Spee

**Affiliations:** Department of Clinical Sciences of Companion Animals, Faculty of Veterinary Medicine, Utrecht University, Utrecht, The Netherlands; TCCI Consultancy BV, Utrecht, The Netherlands

**Keywords:** Laser microdissection, Hepatic progenitor cells, Dog, Wnt/β-catenin, Notch signalling

## Abstract

**Background:**

The liver has a large regenerative capacity. Hepatocytes can replicate and regenerate a diseased liver. However, as is the case in severe liver diseases, this replication may become insufficient or exhausted and hepatic progenitor cells (HPCs) can be activated in an attempt to restore liver function. Due to their bi-potent differentiation capacity, these HPCs have great potential for regenerative approaches yet over-activation does pose potential health risks. Therefore the mechanisms leading to activation must be elucidated prior to safe implementation in the veterinary clinic. Wnt/β-catenin and Notch signalling have been implicated in the activation of HPCs in mouse models and in humans. Here we assessed the involvement in canine HPC activation. Gene-expression profiles were derived from laser microdissected HPC niches from lobular dissecting hepatitis (LDH) and normal liver tissue, with a focus on Wnt/β-catenin and Notch signalling. Immunohistochemical and immunofluorescent studies were combined to assess the role of the pathways in HPCs during LDH.

**Results:**

Gene-expression confirmed higher expression of Wnt/β-catenin and Notch pathway components and target genes in activated HPC niches in diseased liver compared to quiescent HPC niches from normal liver. Immunofluorescence confirmed the activation of these pathways in the HPCs during disease. Immunohistochemistry showed proliferating HPCs during LDH, and double immunofluorescence showed downregulation of Wnt/β-catenin and Notch in differentiating HPCs. Vimentin, a mesenchymal marker, was expressed on a subset of undifferentiated HPCs.

**Conclusions:**

Together these studies clearly revealed that both Wnt/β-catenin and Notch signalling pathways are enhanced in undifferentiated, proliferating and potentially migrating HPCs during severe progressive canine liver disease (LDH).

## Background

Liver diseases occur frequently in the canine pet population. Around 12% of the dogs in first opinion practices have liver disease [1,2] and account for 1-2% of a university veterinary clinical population [3]. It is conceivable that these numbers, based on the Cambridge region (UK) and the Utrecht University Clinics (the Netherlands), respectively, are exemplary for the West-European dog pet population. One third of chronic hepatitis cases are caused by copper accumulation. In addition, microorganisms, toxins and drugs have been reported to cause hepatitis in dogs. In more than 60% of cases, however, hepatitis remains idiopathic [3,4]. The recently discovered canine hepacivirus is unlikely to cause canine hepatitis [5-7].

Irrespective of the cause of hepatocyte damage, the liver can recover from such insults due to replication of fully differentiated hepatocytes [8]. In case this replication is exhausted or otherwise hampered, hepatic progenitor cells (HPCs) are reported to have the potential to take over regeneration. These stem cells are believed to be bi-potential and to have the capacity to differentiate into either hepatocytes or cholangiocytes, depending on cellular demand [9]. HPCs are located in the terminal branches of the biliary tree, called the Canal of Hering [10]. Several publications describe the cellular and molecular constituents of the canine or feline HPC niche [11-15]. The niche is not just an anatomical region in the liver, but it has a biological function as it provides the cell- and matrix derived signals to instruct the HPC’s cellular fate.

In order to safely use HPCs for liver regeneration in a clinical setting where hepatocyte replication is insufficient, detailed knowledge of crucial signalling cascades for HPC activation is essential. Two signalling pathways, Wnt/β-catenin and Notch, are involved in proliferation and differentiation of progenitor cells including HPCs in other mammals [16-20]. This prompted the present focus on these two transmembrane signalling pathways in the activation of HPCs in canine liver diseases. Since lobular dissecting hepatitis (LDH) was previously observed to contain the highest number of activated HPCs, the activated HPC niche from LDH was microdissected and molecular analyses were performed in comparison with quiescent HPC niches harboured adjacent to portal areas of normal canine liver [13].

These data show that both Wnt/β-catenin and Notch signalling are enhanced in activated HPC niches in dogs with LDH. The previous descriptions of the cellular constituents of the canine HPC niche and the current investigation on specific signalling cascades clearly show the similarities with other mammals, including human [12,13]. It is therefore conceivable that results of clinical approaches in human medicine will be applicable and beneficial in veterinary health care.

## Results

### Gene-expression profiling of laser microdissected HPC niches indicate activation of Wnt and Notch pathways

**Figure 1:**
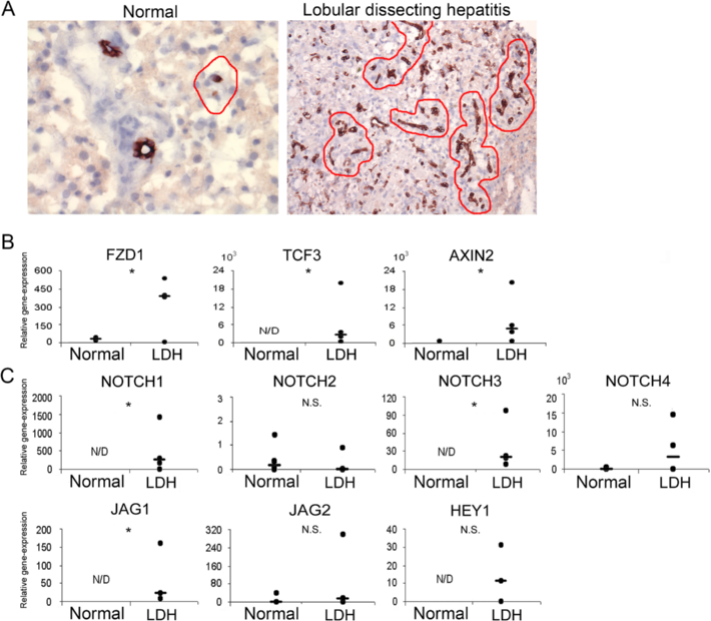
**Gene expression of Wnt and Notch signalling components is enhanced in HPC niches during disease.** Examples of cryosections (6 μm) of normal liver and lobular dissecting hepatitis immunostained for Keratin 7 **(A)**, a marker of HPCs and cholangiocytes. The red lines indicate the quiescent HPC niche in the periportal area of normal tissue and the activated HPC niche throughout the parenchyma of diseased tissue **(A)**. These areas were specifically selected by means of laser microdissection for RNA isolation and gene expression analysis. Relative gene-expression for components of Wnt **(B)**, and Notch **(C)** signalling pathways show upregulation of these pathways in HPC niches during disease. FZD1, Frizzled 1; TCF3, transcription factor 3; AXIN2, axis inhibitor 2; JAG, jagged; HEY1, hairy/enhancer of split-related with YRPW motif; N.S., not significant; N/D, not detectable.

Representative pictures of laser microdissected HPC niches in normal liver and lobular dissecting hepatitis (LDH) stained with Keratin(K)7 (marker of HPCs) are depicted in Figure 1A. Expression levels of the Wnt receptor *FZD1* and the Wnt-induced transcription factor *TCF3* were significantly higher in LDH cases compared to normal controls, as measured in LMD samples (Figure 1B). Of the various Notch-receptor proteins, only *NOTCH1* and *NOTCH3* expression levels were significantly higher in diseased material (Figure 1C). In line is the observation that only ligand *JAG1* is upregulated, whereas *JAG2* is not (Figure 1C). Based on these expression levels of ligand and receptors, it was anticipated that an activated Wnt/β-catenin and Notch signalling cascade would be present in activated HPC niches (Figure 1B,C). Importantly, the expression levels of classical target genes for Wnt/β-catenin, *AXIN2,* and Notch signalling, *HEY1*, were indeed elevated in LDH, confirming active downstream signalling (Figure 1B,C).

### Immunofluorescence confirm activated Wnt/β-catenin and Notch signalling in HPC during disease

**Figure 2:**
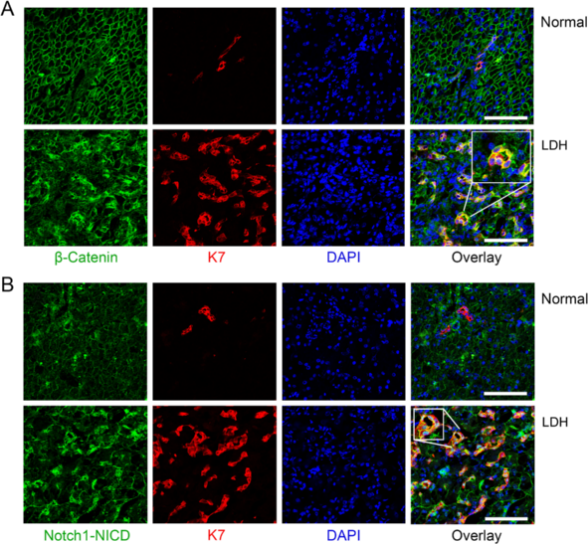
**Wnt and Notch signalling are active in HPCs during disease.** Example of immunofluorescent double staining on cryosections (15 μm) against β-catenin (green) and Keratin(K)7 (red), with ToPro3 (blue) nuclear counterstaining in normal and LDH liver tissue **(A**, size bar indicates 100 μm). In normal tissue, β-catenin is present in a membranous staining pattern on hepatocytes and bile duct cells. No nuclear β-catenin was seen. In LDH, β-catenin is clearly increased and overlay shows cytoplasmic and nuclear presence of β-catenin in K7 positive cells (yellow and bright blue). Example of immunofluorescent double staining on cryosections (15 μm) against Notch/Notch Intra Cellular Domain (Notch/NICD; green) and K7 (red), with ToPro3 (blue) nuclear counterstaining in normal and diseased (LDH) liver tissue **(B**, size bar indicates 100 μm). In normal tissue, a canalicular staining pattern on hepatocytes and bile duct cells was found, no nuclear staining was present.

To specify the cellular origin of the upregulated gene expression levels, double immunofluorescence for β-catenin/K7 (Figure 2A) and Notch1/Notch Intra Cellular Domain (NICD)/K7 was performed (Figure 2B). This revealed that β-catenin and Notch1/NICD were strongly expressed in the cytoplasm and/or nucleus of the cells of the ductular reaction in LDH. Expression of β-catenin in hepatocytes and cholangiocytes was membranous, indicative for a low activation status of the Wnt/β-catenin signalling cascade. Similarly, Notch1/NICD was expressed in canalicular- and less pronounced in basolateral membranes in hepatocytes in normal tissues.

### Wnt/β-catenin and Notch signalling coincide with HPC proliferation and mesenchymal characteristics but are lost with differentiation

**Figure 3:**
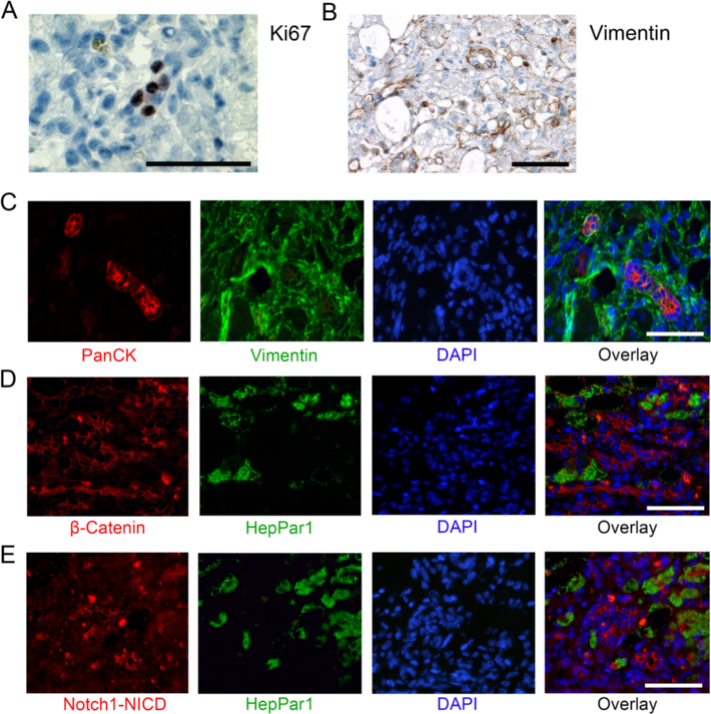
**Proliferation and mesenchymal characteristics on HPCs and relation of differentiation with β-catenin and Notch1/NICD signalling.** Immunohistochemical staining for Ki67 shows positive cells in the ductular reaction during LDH **(A)**. Immunohistochemical staining for vimentin suggests positive ductular reactions **(B)** and an example of vimentin and PanCK double staining **(C)** shows clear co-localisation on HPCs in LDH. Double immunofluorescence against HepPar1 and β-catenin **(D)** or Notch1/Notch intracellular domain (Notch1/NICD; **(E)** in LDH shows polarisation of the ductular reaction: clear cytoplasmic staining of β-catenin or Notch/NICD is present in non-differentiated cells of the ductular reaction, and only membranous staining is present in differentiating and fully differentiated, HepPar1 positive, hepatocytes. Size bars indicate 50 μm.

To investigate the functional involvement of Wnt and Notch in HPC activation, immunohistochemical and immunofluorescent stainings were performed (Figure 3). With Ki67 staining the proliferative activity in the tissues was assessed. In LDH, very few hepatocytes were found to be positive for Ki67, while a substantial number of Ki67 positive cells were found in the DR (Figure 3A). To evaluate the potential of HPCs to obtain mesenchymal characteristics, immunohistochemical staining for vimentin and double immunofluorescent staining for vimentin and PanCK was performed (Figure 3B,C). This revealed strong (co-)staining of vimentin in ductular structures, clearly showing expression of a mesenchymal marker on HPCs. To investigate the role of Wnt and Notch in HPC differentiation during liver disease, the mature hepatocyte marker HepPar1 in combination with β-catenin or Notch1/NICD in a double immunofluorescent staining was used. A clear polarisation of the ductular reaction was observed in such a way that the non-differentiated cells exhibited strong cytoplasmic and sometimes nuclear staining for both β-catenin and Notch1/NICD. This staining was lost in the intermediate and HepPar1 positive differentiated hepatocytes during continuation of the ductular reaction (Figure 3D,E). This is in line with the cytoplasmic and nuclear staining of β-catenin and Notch1/NICD in the K7-positive undifferentiated HPCs in Figure 2. In all staining procedures, negative controls remained negative, indicating the specificity of the antibodies used.

## Discussion

In the present study the involvement of the Wnt/β-catenin and Notch pathways was investigated in canine hepatic progenitor cell activation in LDH, a highly fibrotic and progressive liver disease. The combination of laser-microdissection, gene expression studies (Q-PCR) and immunofluorescence showed the enhanced signalling of the Wnt/β-catenin and Notch pathways in activated HPC niches compared to quiescent HPCs in normal liver. This extends the findings in mouse and human liver cancer and normal liver regeneration [19,21-27]. The fact that this apparent activation was insufficient to restore the liver, dogs presenting with LDH die within a year after diagnosis [4], suggests that therapeutic opportunities are present here. The reason why HPCs fail to regenerate the liver seems to vary with the type of disease and may be due to insufficient proliferation, migration or differentiation [28]. New therapeutic strategies could address the pathways involved in these phases of activation.

The effect of Wnt/β-catenin was previously specified as (induction of) proliferation in rodent models of HPC activation [16,24,29,30]. Similarly, a regulatory role for Notch in HPC proliferation is described [31,32]. In LDH, the concurrent presence of active Wnt and Notch signalling and actively proliferating, Ki67 positive cells in the ductular reaction suggest a potential role for these pathways in proliferation of canine HPCs during disease as well. Another functional implication of Wnt and Notch signalling may relate to the acquisition of mesenchymal characteristics by HPCs. The presence of mesenchymal characteristics can relate to migratory potential, which is not equivalent to full epithelial-mesenchymal transition (EMT), a disputed phenomenon in adult liver [33-35]. From a regenerative point of view, migration is necessary for HPCs to move toward the site of disease activity, and is likely to occur in concert with proliferation [36,37]. Wnt and Notch pathways have been implicated in EMT and migratory potential of cells in different types of tissues and cancer development [38,39]. In canine LDH vimentin positive HPCs also exhibit Wnt and Notch signalling, potentially indicating a causative relation in canine HPCs. It will be of interest to further functionally investigate this newly suggested role of Wnt or Notch in migration of HPCs during disease, as it may provide interesting potential for therapeutic intervention.

Besides the described role in proliferation and migration, both pathways can be involved in HPC differentiation. The influence of Wnt on cell fate determination is time and place dependent. During early embryonic development and in pluripotent embryonic stem cells *in vitro* Wnt activation leads to hepatic specification [40,41]. Later in foetal liver development and *in vitro* in more committed multipotent cells, active Wnt inhibits (further) hepatocyte differentiation but rather guides cells to the biliary phenotype [42,43]. Regarding the HPC as a committed progenitor cell, Wnt activation in LDH might stimulate bile duct differentiation and inhibit hepatocyte differentiation. An interesting finding in this study is that small hepatocytes lying in continuation with ductular cells, and possibly representing intermediate hepatocytes [44], display a membranous β-catenin staining pattern (Figure 3) similar to that of hepatocytes in normal tissue. This supports the theory that the Wnt/β-catenin pathway is no longer active during hepatocytic differentiation of ductular cells and is different from previous mouse data [21]. Unfortunately, the lack of specific markers for intermediate hepatocytes limits their description to size and localization only [11,45]. The importance of Notch in liver development and hepatocyte differentiation is apparent in the mutation in the Notch ligand Jag1, which is associated with Alagille syndrome, presenting with aberrant bile duct development [25,46-51]. More recently a distinctive role for the different Notch receptors has been explored, suggesting that Jag1-mediated Notch1 and Notch3 activation stimulates differentiation of hepatoblasts towards the biliary phenotype and inhibits hepatocytic differentiation. Vice-versa, Notch1 and Notch3 expression would be lost when (liver progenitor) cells differentiated towards hepatocytes [52,53]. Converting these findings to the described results, it might be postulated that during LDH, where *NOTCH1* and *NOTCH3* expression is increased, HPC differentiation towards hepatocytes is inhibited, while bile duct differentiation may be enhanced. This is corroborated by the immunofluorescence stainings, where Notch1/NICD is lost with differentiation.

The activated states of the Wnt and Notch pathway in the diseased tissue were found at the same histological location, suggesting that Wnt and Notch act simultaneously. In view of this, the Wnt and Notch pathways may be intertwined in the activation of HPCs during liver disease, as occurs for example when cell-fate decisions are made during development [54]. Whether and how Wnt and Notch interact during HPC activation and in what manner this can be used for therapeutic benefit needs to be further investigated in molecular *in vitro* studies.

## Conclusions

The combined Q-PCR and immunofluorescence results extend existing literature on other species and indicate a critical role for Wnt and Notch in proliferation, differentiation and/or migration of canine HPCs during rapidly progressing fibrotic liver disease with hampered hepatocytic proliferation. The descriptive data presented here suggest a role in HPC activation; *in vitro* experiments with canine hepatic progenitor cells, at present not available, could shed light on these questions separately. These data from a non-experimental liver disease in client-owned pets confirm the previous separate reports on Wnt and Notch signalling in rat and mouse injury models of liver disease [16,23,24,29,30,50] and human data [19]. In the future, the implementation of pre-clinical experiments with Notch or Wnt inhibitors in order to enhance liver regeneration in patients could be mutually beneficial for dog and man.

## Methods

### Liver samples

Liver samples were obtained from privately owned dogs with LDH (n = 4; age range 1.5-2.5 years), a rapidly progressing disease characterized by diffuse inflammation, peri-cellular fibrosis and massive HPC activation [12,13]. Informed consent was obtained as required under Dutch legislation. Liver pathology of the dogs was confirmed histologically by one board-certified veterinary pathologist according to the World Small Animal Veterinary Association (WSAVA)-standards [55]. Normal livers (n = 4; age range 1-3 years) were obtained from surplus animals of a non-liver related research project at the University Medical Centre Utrecht (University 3R policy).

### Laser microdissection (LMD) of Keratin(K)7 positive cell patterns

Cryosections (10 μm) were cut using RNAse free blades on a cryostat at -20°C (Leica CM3050 cryostat, Leica Microsystems GmbH, Wetzlar, Germany), mounted on a pre-cooled (4°C) RNAse-free poly-ethylene naphthalene (PEN) membrane slide (P.A.L.M. MicroLaser Technologies AG, Burnried, Germany), immediately placed on dry ice and stored at -70°C for a maximum of one week until use. Rapid immunohistochemistry before laser microdissection (LMD) was performed with buffers and solutions prepared with DEPC-treated water (Ambion, Austin, TX). All glassware was treated with RNase Zap (Ambion) and washed with DEPC-treated water prior to use. To protect RNA from degradation during incubations at room temperature (RT), antibody and DAB solutions were prepared with 0.4 U/μl SUPERase RNase Inhibitor (Ambion). Frozen sections were taken from -70°C storage, immediately fixed in ice-cold acetone (-20°C) for 5 minutes and washed briefly (3-5 seconds) in phosphate-buffered saline (PBS). Sections were incubated with K7 antibody (1:20; Dako, Glostrup, Denmark) in PBS for 7 minutes at RT, briefly washed in PBS and subsequently incubated in EnVision goat anti-mouse peroxidase-conjugated antibody (Dako) for 7 minutes at RT. Staining was visualised using the chromogen diaminobenzidine (DAB; Dako) for 3 minutes at RT. Finally, sections were dehydrated in an EtOH series (75-95-100%, 15 seconds each). The LMD procedure was performed within a maximum of 20 minutes upon staining with a Nikon eclipse TE300 inverted microscope (Nikon Inc. Instrument Group, Melville, NY) connected to a Sony 3-CCD Microscope ColorColour Video Camera (Sony Electronics Inc., Tokyo, Japan) using MMI CellTools software (MMI Molecular Machines & Industries AG, Glattbrugg, Switzerland). Tubes with an adhesive lid (MMI) were used to remove laser dissected cells from the whole liver tissue slide. After the LMD procedure, collected cells were retrieved with 50 μl Extraction Buffer (PicoPure RNA isolation kit, Molecular Devices, MDS Analytical Technologies, Sunnyvale, CA) and a short centrifugal step for further molecular processing. The cell-suspension was then incubated at 42°C for 30 min, centrifuged for 2 minutes at 800 × g, snap frozen in liquid nitrogen and stored at -70°C until further use. From each sample, a total of 2-3.5 × 10^6^ μm^2^ tissue was laser-dissected using four tissue sections per normal sample and two tissue sections per diseased sample.

### RNA isolation and amplification

Total RNA was extracted from the LMD samples using the PicoPure RNA isolation kit (MDS Analytical Technologies) according to the manufacturer’s instructions and an on column DNAse treatment (0.1 U/μl) to remove all DNA contaminations (Qiagen, Benelux BV, Venlo, The Netherlands). RNA quality after LMD was determined using a RNA 6000 Pico-LabChip with an Agilent BioAnalyzer 2100 (Agilent, Palo Alto, CA). Amplification of the RNA was performed with the WT-Ovation™ Pico RNA Amplification System according to the manufacturer’s instructions (NuGEN Technologies Inc., Bemmel, The Netherlands). Making use of a DNA/RNA chimeric primer, cDNA was prepared from total RNA and amplified by linear isothermal DNA amplification. The product consists of single-strand DNA (ssDNA). The amplified product was purified with DNA Clean & Concentrator-25 from Zymo research according to the manufacturers instruction (Baseclear Lab Products, Leiden, The Netherlands), replacing Wash Buffer by fresh 80% ethanol.

### Q-PCR analysis

For gene expression analysis, a SYBR Green based quantitative RT-PCR (Q-PCR) was performed on a Bio-Rad My-IQ detection system as described previously [56] for up to 45 cycles. Gene expression of described markers of HPCs and hepatocytes and of components of the Wnt/β-catenin and Notch signalling pathways was measured. Details of the primers and PCR conditions are listed in Table 1. Sequencing reactions confirmed amplification of the specific primer products in the Q-PCR reaction. Normalisation was secured due to the use of at least three independent reference genes: *B2MG*, *HPRT*, *RPS5*, and *RPS19*.

**Table 1:** **Primers and PCR conditions**

**Gene**	**Direction**	**Sequence (5’- 3’)**	**Tm (°C)**	**Product size (bp)**	**Genbank accession number**
B2MG^a^	Forward	TCCTCATCCTCCTCGCT	60.3	85	XM_535458
	Reverse	TTCTCTGCTGGGTGTCG			
HPRT^a^	Forward	AGCTTGCTGGTGAAAAGGAC	58	114	NM_001003357
	Reverse	TTATAGTCAAGGGCATATCC			
RPS5^a^	Forward	TCACTGGTGAGAACCCCCT	62.5	141	XM_533568
	Reverse	CCTGATTCACACGGCGTAG			
RPS19^a^	Forward	CCTTCCTCAAAAAGTCTGGG	61	95	XM_533657
	Reverse	GTTCTCATCGTAGGGAGCAAG			
FZD1	Forward	GGCGCAGGGCACCAAGAAG	58.8	97	XM_539411
	Reverse	GAGCGACAGAATCACCCACCAGA			
TCF3	Forward	GGTGAATGAGCGGGTCCTGAACA	58.8	128	XM_849145
	Reverse	TGAGCTGGCTGGCACGGTAGTC			
AXIN2	Forward	CACCCGCTCTACAACAAGGT	60	128	XM_548025
	Reverse	AGGTGGAGATGAAGCACAGC			
NOTCH1	Forward	TACCGGCCAGAACTGTGAGGAGAA	56	108	XM_537795
	Reverse	GGAGGGCAGCGGCAGTTGTAAGTA			
NOTCH2	Forward	AGCACGCATCCTGGCATACCTC	58.3	106	XM_853135
	Reverse	TGGGGATTAGCTGGAAAGTCACAA			
NOTCH3	Forward	TCTGCCAGAGTTCCGTGGTG	66.8	117	XM_847948
	Reverse	ATGGGGTACAAGGGCTGCTG			
NOTCH4	Forward	GGAAGGGAGCCAGGGACCAACACA	68	96	NM_004557
	Reverse	TCAGGGCCACAGCGGGACAAATC			
JAG1	Forward	GGGCAACACCTTCAATCTCAAG	58.5	122	XM_853730
	Reverse	CATTACTGGAATCCCACGCTTC			
JAG2	Forward	GGGTACGTGCGTGGGC	64		XM_548004
	Reverse	CACCGTTGTAGCAAGGCAG			
HEY1	Forward	CCAGGAAAAGACGAAGAGGC	62.5	226	NM_001002953
	Reverse	CTCCGATAGTCCATAGCAAGGG			

Table legend text. ^a^Reference genes.

### Statistical analysis

Relative gene expression of each gene-product (delta-Cq method) was used as the basis for all comparisons. The non-parametric Mann-Whitney U test was performed to assess statistical differences between normal and diseased tissue using SPSS software (SPSS Benelux, Gorinchem, the Netherlands). Gene expressions that were not detectable were arbitrarily set to Cq 45 for statistical analysis. p-Values < 0.05 were considered statistically significant.

### Immunohistochemistry/-fluorescence

Antibody details for immunohistochemistry/-fluorescence can be found in Table 2.

**Table 2:** **Antibody specifications**

	**Source/type**	**Clone**	**Company**	**Antigen retrieval**	**Dilution**
β-Catenin	Rb/po		Abcam	TE pH9	1:2,000
HepPar1	Mu/mo		Dako	TE pH9	1:50
K7	Mu/mo	OV-TL 12/30	Dako		1:50
Ki67	Rb/mo	SP6	LabVision		1:50
PanCK	Rb/po		Dako	TE pH9	1:400
Notch1/NICD	Rb/po	C-20	Santa Cruz	TE pH9	1:100
Vimentin	Mu/mo		Abcam	TE pH9	1:200

Mu: Mouse; Rb: Rabbit; Mo: monoclonal; Po: polyclonal. TE: Tris/EDTA.

Whole liver cryosections (6 μm) were immunohistochemically stained for Ki67, a marker of active cell proliferation. Slides were air dried for 30 minutes at RT, fixed in ice-cold aceton:methanol 1:1 and washed in phosphate buffered saline with 0.1% Tween 20 (PBS/T, pH 7.4). Endogenous peroxidase activity was blocked for 30 minutes at RT in 0.3% H_2_O_2_ in PBS/T and background staining was blocked with 10% normal goat serum in PBS/T for 30 minutes at RT. Primary antibody was diluted in blocking serum and incubated for 60 minutes at RT. The HRP Envision system (Dako) was used. Staining was visualised using diaminobenzidine (DAB; Dako) and counter stained with haematoxylin quickstain (Vector Laboratories) for 5 minutes. Finally, slides were covered with Aquamount (Vector Laboratories). Canine duodenum served as the positive control (data not shown). Immunohistochemistry for vimentin was performed on 4 μm thick, paraffin embedded sections essentially as described previously [13]. Immunohistochemical pictures were obtained using an Olympus BX41TF Microscope (Olympus Corporation, Tokyo, Japan) with an Olympus U-CMAD3 camera and CellˆB software (AnalySIS, Olympus).

Immunofluorescent stainings were performed with parallel antibody incubations. The slides were incubated with mixed primary antibodies over night at 4°C and with mixed secondary antibodies at RT for 60 minutes. Rinsing steps were performed using TBS with 0.1% Tween 20 and slides were covered with Aquamount (Vector Laboratories). Immunofluorescent double stainings for K7/β-catenin and K7/Notch1-NICD were performed on 15 μm ice-cold acetone fixed (normal and LDH) liver-cryosections. Immunofluorescent double stainings against PanCK/Vimentin, HepPar/β-catenin and HepPar/Notch1-NICD were performed on 4 μm paraffin embedded canine LDH liver sections essentially as described previously [13]. For Notch1-NICD, incubation in 0.5% Triton for 20 minutes at RT was included for permeabilisation. The nucleus was stained with ToPro3 or DAPI. Slides were analysed using a Leica TCS SPE-II Confocal microscope and Leica software. In all procedures, negative controls were included constituting of a bilateral isotype control.
